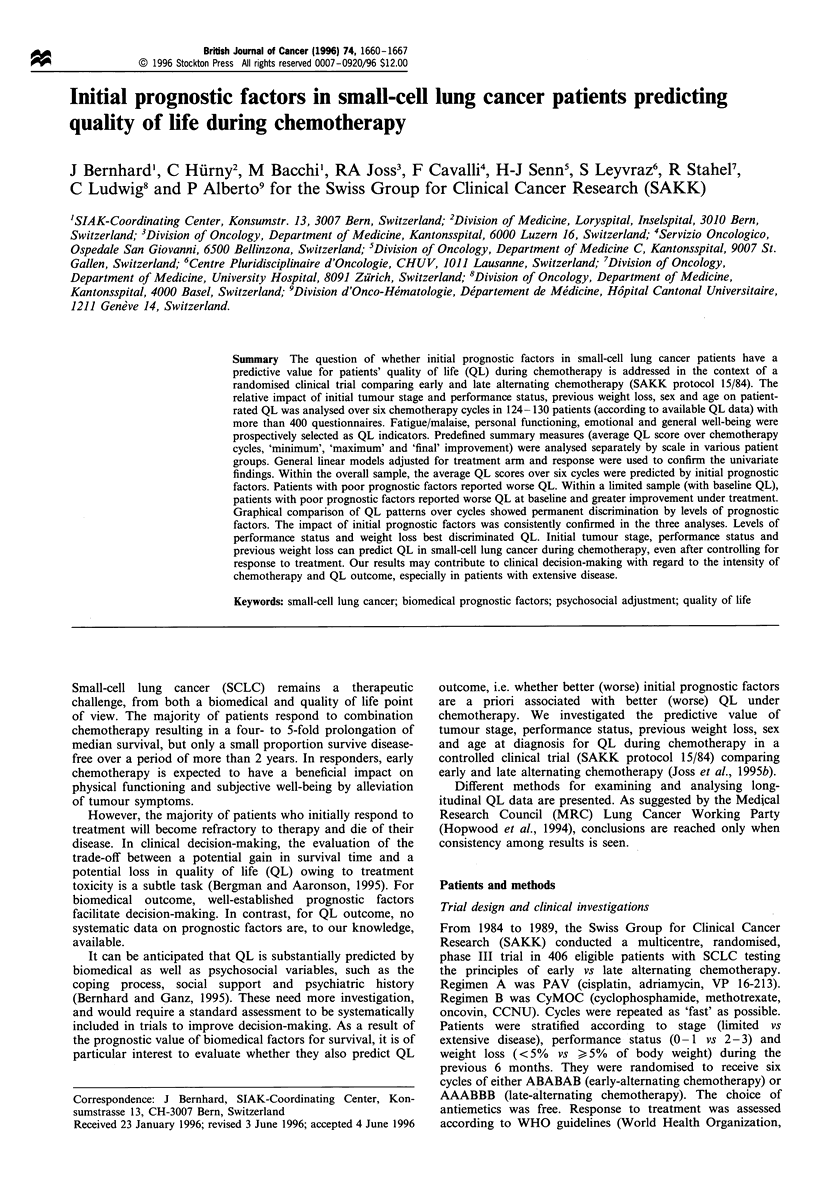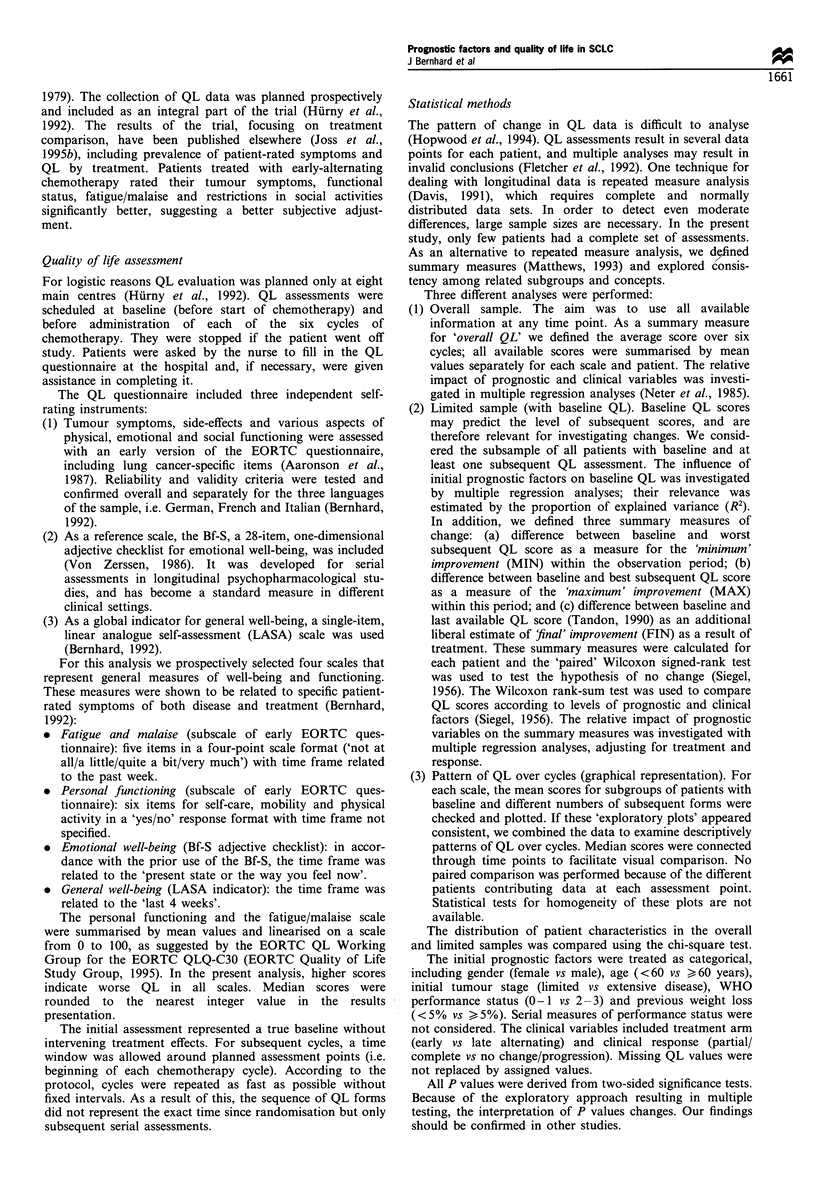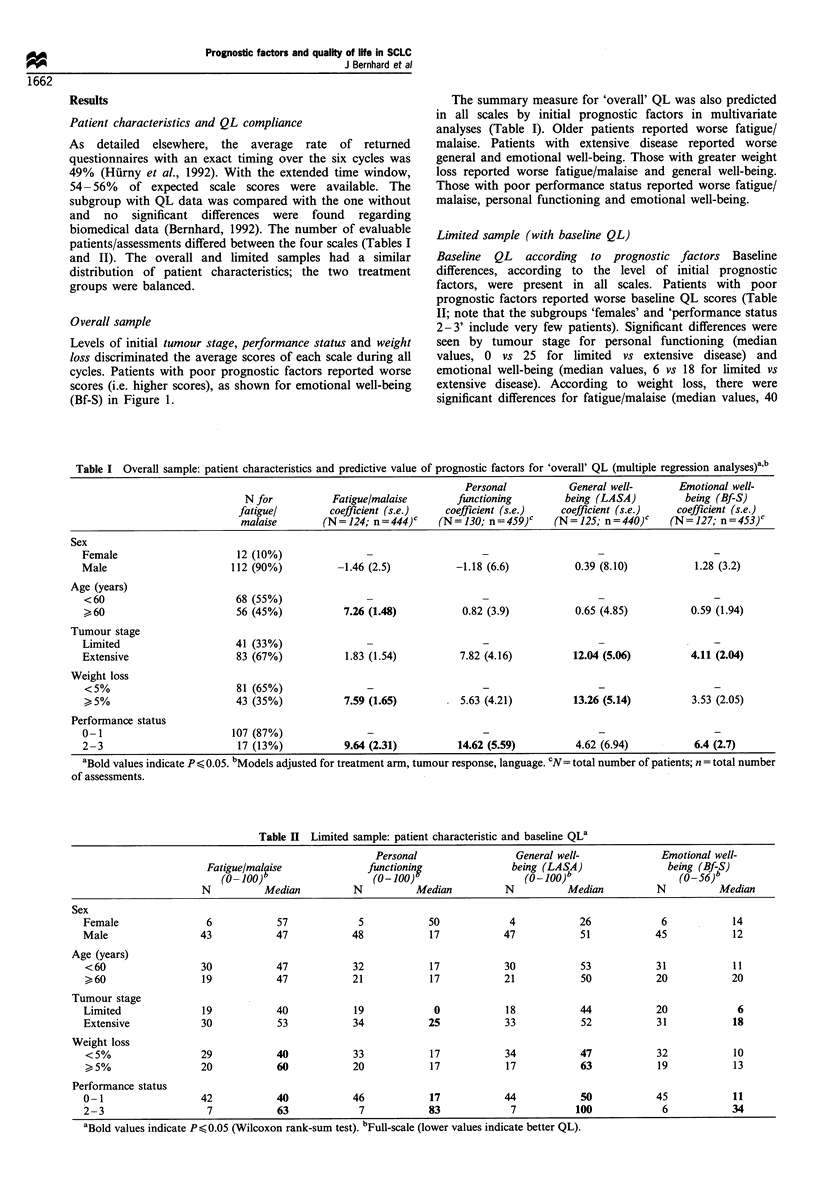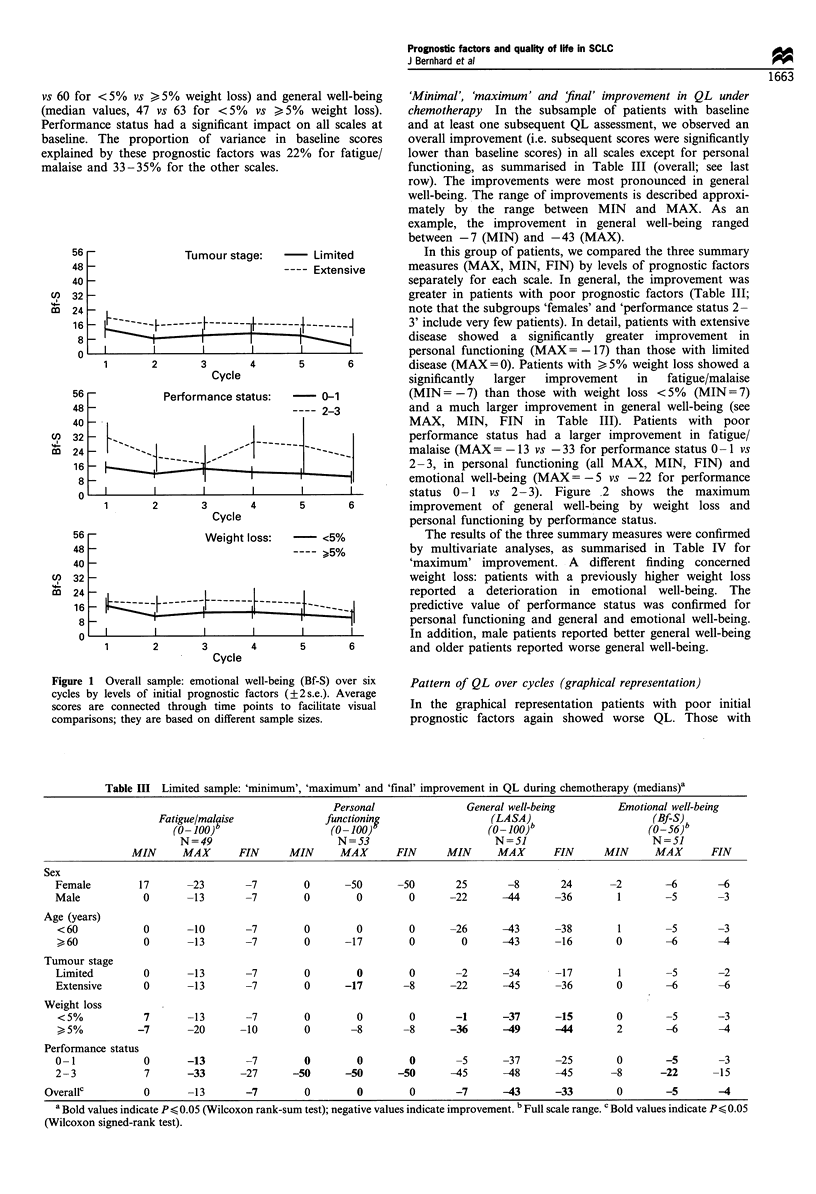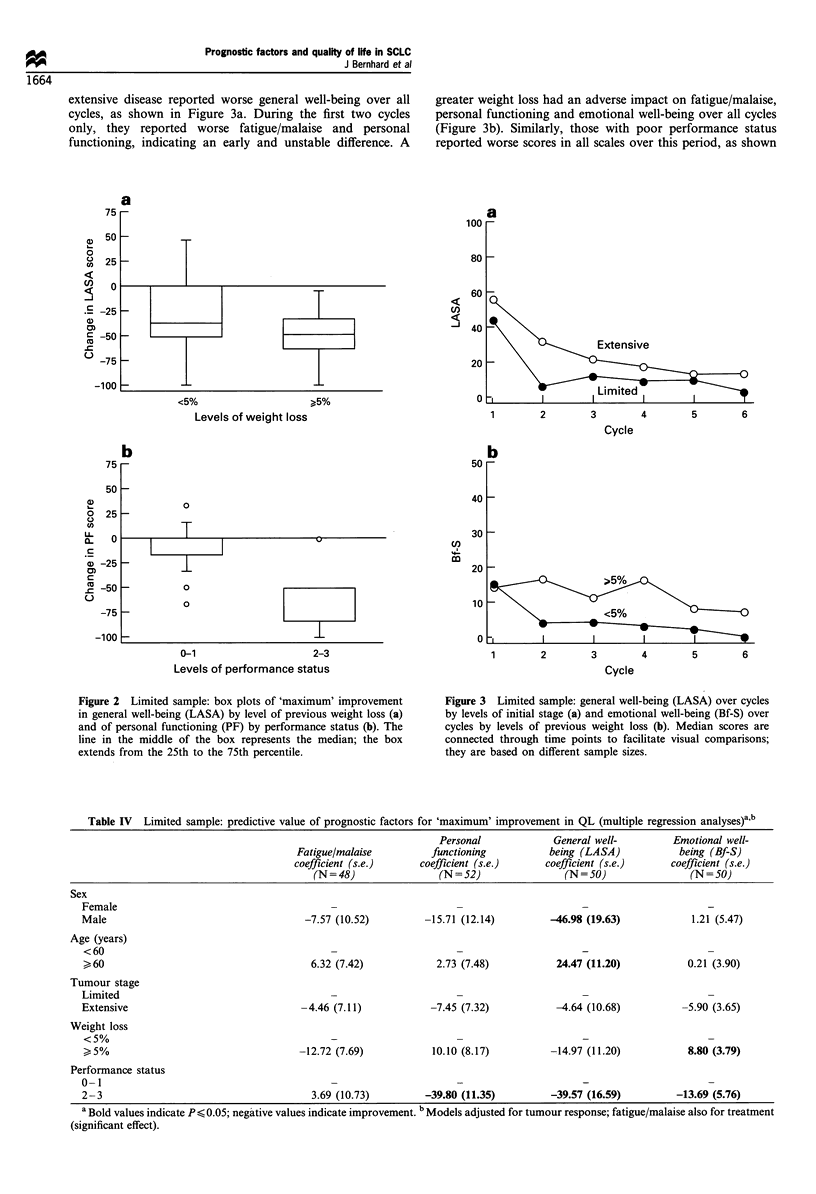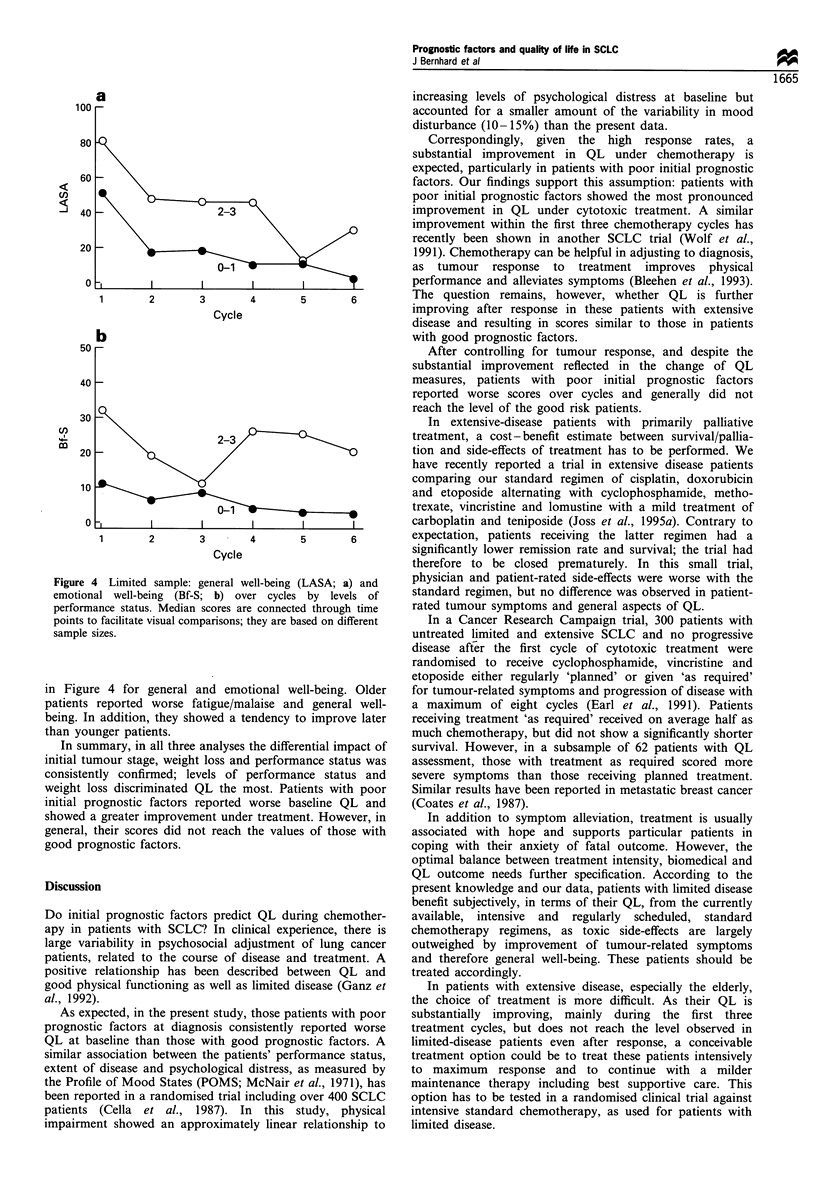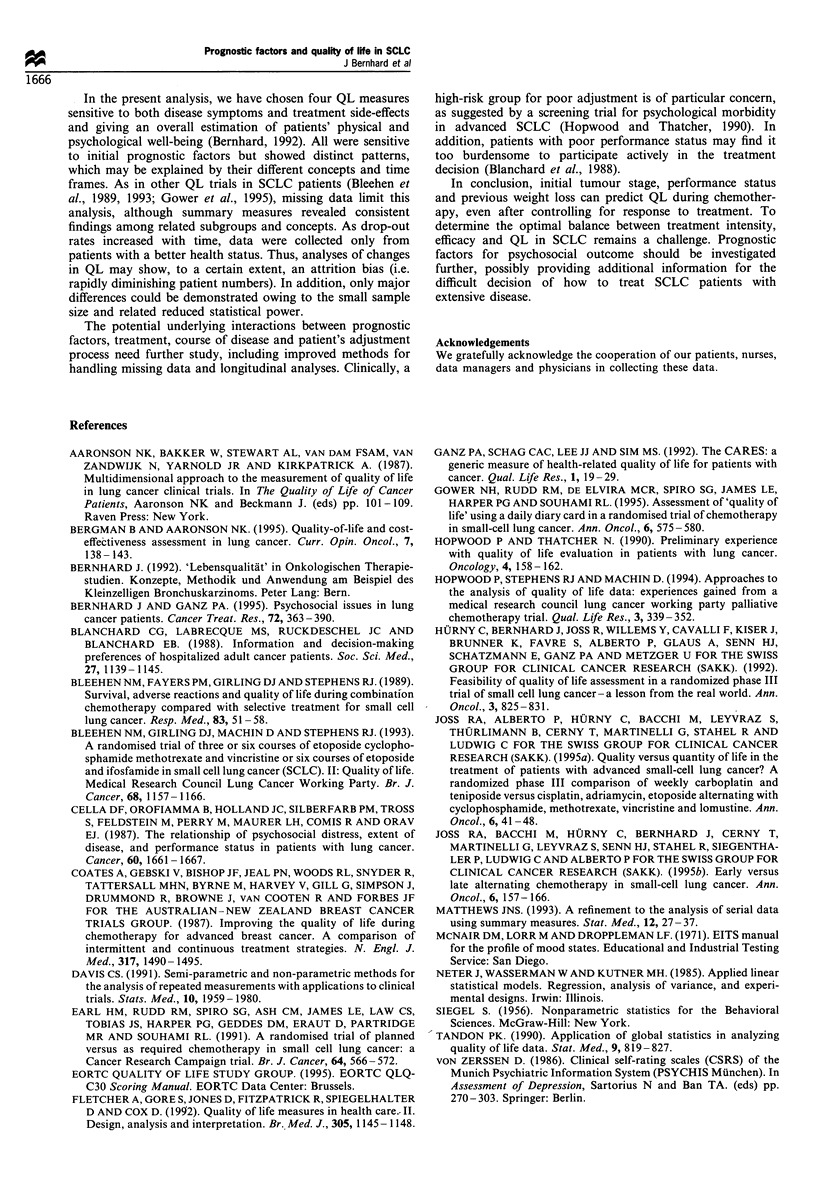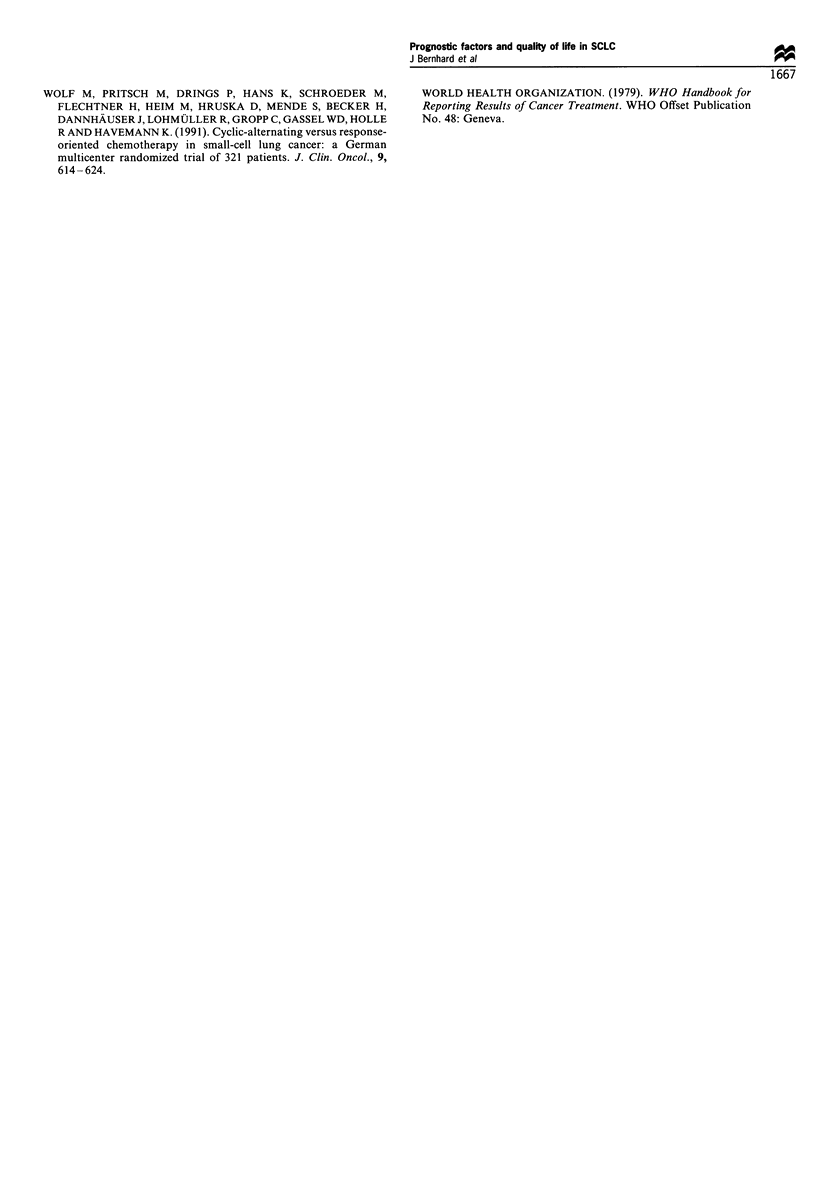# Initial prognostic factors in small-cell lung cancer patients predicting quality of life during chemotherapy. Swiss Group for Clinical Cancer Research (SAKK).

**DOI:** 10.1038/bjc.1996.606

**Published:** 1996-11

**Authors:** J. Bernhard, C. Hürny, M. Bacchi, R. A. Joss, F. Cavalli, H. J. Senn, S. Leyvraz, R. Stahel, C. Ludwig, P. Alberto

**Affiliations:** SIAK-Coordinating Center, Bern, Switzerland.

## Abstract

The question of whether initial prognostic factors in small-cell lung cancer patients have a predictive value for patients' quality of life (QL) during chemotherapy is addressed in the context of a randomised clinical trial comparing early and late alternating chemotherapy (SAKK protocol 15/84). The relative impact of initial tumour stage and performance status, previous weight loss, sex and age on patient-rated QL was analysed over six chemotherapy cycles in 124-130 patients (according to available QL data) with more than 400 questionnaires. Fatigue/malaise, personal functioning, emotional and general well-being were prospectively selected as QL indicators. Predefined summary measures (average QL score over chemotherapy cycles, 'minimum', 'maximum' and 'final' improvement) were analysed separately by scale in various patient groups. General linear models adjusted for treatment arm and response were used to confirm the univariate findings. Within the overall sample, the average QL scores over six cycles were predicted by initial prognostic factors. Patients with poor prognostic factors reported worse QL. Within a limited sample (with baseline QL), patients with poor prognostic factors reported worse QL at baseline and greater improvement under treatment. Graphical comparison of QL patterns over cycles showed permanent discrimination by levels of prognostic factors. The impact of initial prognostic factors was consistently confirmed in the three analyses. Levels of performance status and weight loss best discriminated QL. Initial tumour stage, performance status and previous weight loss can predict QL in small-cell lung cancer during chemotherapy, even after controlling for response to treatment. Our results may contribute to clinical decision-making with regard to the intensity of chemotherapy and QL outcome, especially in patients with extensive disease.


British Journal of Cancer (1996) 74, 1660-1667
© 1996 Stockton Press All rights reserved 0007-0920/96 $12.00

### Initial prognostic factors in small-cell lung cancer patients predicting quality of life during chemotherapy

J Bernhard[1], C Hürny[2], M Bacchi[1], RA Joss[3], F Cavalli[4], H-J Senn[5], S Leyvraz[6], R Stahel[7], C Ludwig[8] and P Alberto[9] for the Swiss Group for Clinical Cancer Research (SAKK)

*[1]SIAK-Coordinating Center, Konsumstr. 13, 3007 Bern, Switzerland; [2]Division of Medicine, Loryspital, Inselspital, 3010 Bern, Switzerland; [3]Division of Oncology, Department of Medicine, Kantonsspital, 6000 Luzern 16, Switzerland; [4]Servizio Oncologico, Ospedale San Giovanni, 6500 Bellinzona, Switzerland; [5]Division of Oncology, Department of Medicine C, Kantonsspital, 9007 St. Gallen, Switzerland; [6]Centre Pluridisciplinaire d'Oncologie, CHUV, 1011 Lausanne, Switzerland; [7]Division of Oncology, Department of Medicine, University Hospital, 8091 Zürich, Switzerland; [8]Division of Oncology, Department of Medicine, Kantonsspital, 4000 Basel, Switzerland; [9]Division d'Onco-Hématologie, Département de Médicine, Hôpital Cantonal Universitaire, 1211 Genève 14, Switzerland.*

**Summary** The question of whether initial prognostic factors in small-cell lung cancer patients have a predictive value for patients' quality of life (QL) during chemotherapy is addressed in the context of a randomised clinical trial comparing early and late alternating chemotherapy (SAKK protocol 15/84). The relative impact of initial tumour stage and performance status, previous weight loss, sex and age on patient-rated QL was analysed over six chemotherapy cycles in 124-130 patients (according to available QL data) with more than 400 questionnaires. Fatigue/malaise, personal functioning, emotional and general well-being were prospectively selected as QL indicators. Predefined summary measures (average QL score over chemotherapy cycles, 'minimum', 'maximum' and 'final' improvement) were analysed separately by scale in various patient groups. General linear models adjusted for treatment arm and response were used to confirm the univariate findings. Within the overall sample, the average QL scores over six cycles were predicted by initial prognostic factors. Patients with poor prognostic factors reported worse QL. Within a limited sample (with baseline QL), patients with poor prognostic factors reported worse QL at baseline and greater improvement under treatment. Graphical comparison of QL patterns over cycles showed permanent discrimination by levels of prognostic factors. The impact of initial prognostic factors was consistently confirmed in the three analyses. Levels of performance status and weight loss best discriminated QL. Initial tumour stage, performance status and previous weight loss can predict QL in small-cell lung cancer during chemotherapy, even after controlling for response to treatment. Our results may contribute to clinical decision-making with regard to the intensity of chemotherapy and QL outcome, especially in patients with extensive disease.

**Keywords:** small-cell lung cancer; biomedical prognostic factors; psychosocial adjustment; quality of life

Small-cell lung cancer (SCLC) remains a therapeutic challenge, from both a biomedical and quality of life point of view. The majority of patients respond to combination chemotherapy resulting in a four- to 5-fold prolongation of median survival, but only a small proportion survive disease-free over a period of more than 2 years. In responders, early chemotherapy is expected to have a beneficial impact on physical functioning and subjective well-being by alleviation of tumour symptoms.

However, the majority of patients who initially respond to treatment will become refractory to therapy and die of their disease. In clinical decision-making, the evaluation of the trade-off between a potential gain in survival time and a potential loss in quality of life (QL) owing to treatment toxicity is a subtle task (Bergman and Aaronson, 1995). For biomedical outcome, well-established prognostic factors facilitate decision-making. In contrast, for QL outcome, no systematic data on prognostic factors are, to our knowledge, available.

It can be anticipated that QL is substantially predicted by biomedical as well as psychosocial variables, such as the coping process, social support and psychiatric history (Bernhard and Ganz, 1995). These need more investigation, and would require a standard assessment to be systematically included in trials to improve decision-making. As a result of the prognostic value of biomedical factors for survival, it is of particular interest to evaluate whether they also predict QL outcome, i.e. whether better (worse) initial prognostic factors are a priori associated with better (worse) QL under chemotherapy. We investigated the predictive value of tumour stage, performance status, previous weight loss, sex and age at diagnosis for QL during chemotherapy in a controlled clinical trial (SAKK protocol 15/84) comparing early and late alternating chemotherapy (Joss *et al.*, 1995*b*).

Different methods for examining and analysing longitudinal QL data are presented. As suggested by the Medical Research Council (MRC) Lung Cancer Working Party (Hopwood *et al.*, 1994), conclusions are reached only when consistency among results is seen.

### Patients and methods

### *Trial design and clinical investigations*

From 1984 to 1989, the Swiss Group for Clinical Cancer Research (SAKK) conducted a multicentre, randomised, phase III trial in 406 eligible patients with SCLC testing the principles of early *vs* late alternating chemotherapy. Regimen A was PAV (cisplatin, adriamycin, VP 16-213). Regimen B was CyMOC (cyclophosphamide, methotrexate, oncovin, CCNU). Cycles were repeated as 'fast' as possible. Patients were stratified according to stage (limited *vs* extensive disease), performance status (0-1 *vs* 2-3) and weight loss (<5% *vs* ≥5% of body weight) during the previous 6 months. They were randomised to receive six cycles of either ABABAB (early-alternating chemotherapy) or AAABBB (late-alternating chemotherapy). The choice of antiemetics was free. Response to treatment was assessed according to WHO guidelines (World Health Organization,

Correspondence: J Bernhard, SIAK-Coordinating Center, Konsumstrasse 13, CH-3007 Bern, Switzerland
Received 23 January 1996; revised 3 June 1996; accepted 4 June 1996

1979). The collection of QL data was planned prospectively and included as an integral part of the trial (Hürny *et al.*, 1992). The results of the trial, focusing on treatment comparison, have been published elsewhere (Joss *et al.*, 1995*b*), including prevalence of patient-rated symptoms and QL by treatment. Patients treated with early-alternating chemotherapy rated their tumour symptoms, functional status, fatigue/malaise and restrictions in social activities significantly better, suggesting a better subjective adjustment.

*Quality of life assessment*

For logistic reasons QL evaluation was planned only at eight main centres (Hürny *et al.*, 1992). QL assessments were scheduled at baseline (before start of chemotherapy) and before administration of each of the six cycles of chemotherapy. They were stopped if the patient went off study. Patients were asked by the nurse to fill in the QL questionnaire at the hospital and, if necessary, were given assistance in completing it.

The QL questionnaire included three independent self-rating instruments:

(1) Tumour symptoms, side-effects and various aspects of physical, emotional and social functioning were assessed with an early version of the EORTC questionnaire, including lung cancer-specific items (Aaronson *et al.*, 1987). Reliability and validity criteria were tested and confirmed overall and separately for the three languages of the sample, i.e. German, French and Italian (Bernhard, 1992).
(2) As a reference scale, the Bf-S, a 28-item, one-dimensional adjective checklist for emotional well-being, was included (Von Zerssen, 1986). It was developed for serial assessments in longitudinal psychopharmacological studies, and has become a standard measure in different clinical settings.
(3) As a global indicator for general well-being, a single-item, linear analogue self-assessment (LASA) scale was used (Bernhard, 1992).

For this analysis we prospectively selected four scales that represent general measures of well-being and functioning. These measures were shown to be related to specific patient-rated symptoms of both disease and treatment (Bernhard, 1992):

- *Fatigue and malaise* (subscale of early EORTC questionnaire): five items in a four-point scale format ('not at all/a little/quite a bit/very much') with time frame related to the past week.
- *Personal functioning* (subscale of early EORTC questionnaire): six items for self-care, mobility and physical activity in a 'yes/no' response format with time frame not specified.
- *Emotional well-being* (Bf-S adjective checklist): in accordance with the prior use of the Bf-S, the time frame was related to the 'present state or the way you feel now'.
- *General well-being* (LASA indicator): the time frame was related to the 'last 4 weeks'.

The personal functioning and the fatigue/malaise scale were summarised by mean values and linearised on a scale from 0 to 100, as suggested by the EORTC QL Working Group for the EORTC QLQ-C30 (EORTC Quality of Life Study Group, 1995). In the present analysis, higher scores indicate worse QL in all scales. Median scores were rounded to the nearest integer value in the results presentation.

The initial assessment represented a true baseline without intervening treatment effects. For subsequent cycles, a time window was allowed around planned assessment points (i.e. beginning of each chemotherapy cycle). According to the protocol, cycles were repeated as fast as possible without fixed intervals. As a result of this, the sequence of QL forms did not represent the exact time since randomisation but only subsequent serial assessments.

*Statistical methods*

The pattern of change in QL data is difficult to analyse (Hopwood *et al.*, 1994). QL assessments result in several data points for each patient, and multiple analyses may result in invalid conclusions (Fletcher *et al.*, 1992). One technique for dealing with longitudinal data is repeated measure analysis (Davis, 1991), which requires complete and normally distributed data sets. In order to detect even moderate differences, large sample sizes are necessary. In the present study, only few patients had a complete set of assessments. As an alternative to repeated measure analysis, we defined summary measures (Matthews, 1993) and explored consistency among related subgroups and concepts.

Three different analyses were performed:

(1) Overall sample. The aim was to use all available information at any time point. As a summary measure for '*overall QL*' we defined the average score over six cycles; all available scores were summarised by mean values separately for each scale and patient. The relative impact of prognostic and clinical variables was investigated in multiple regression analyses (Neter *et al.*, 1985).
(2) Limited sample (with baseline QL). Baseline QL scores may predict the level of subsequent scores, and are therefore relevant for investigating changes. We considered the subsample of all patients with baseline and at least one subsequent QL assessment. The influence of initial prognostic factors on baseline QL was investigated by multiple regression analyses; their relevance was estimated by the proportion of explained variance ($R^2$). In addition, we defined three summary measures of change: (a) difference between baseline and worst subsequent QL score as a measure for the *'minimum' improvement* (MIN) within the observation period; (b) difference between baseline and best subsequent QL score as a measure of the *'maximum' improvement* (MAX) within this period; and (c) difference between baseline and last available QL score (Tandon, 1990) as an additional liberal estimate of *'final' improvement* (FIN) as a result of treatment. These summary measures were calculated for each patient and the 'paired' Wilcoxon signed-rank test was used to test the hypothesis of no change (Siegel, 1956). The Wilcoxon rank-sum test was used to compare QL scores according to levels of prognostic and clinical factors (Siegel, 1956). The relative impact of prognostic variables on the summary measures was investigated with multiple regression analyses, adjusting for treatment and response.
(3) Pattern of QL over cycles (graphical representation). For each scale, the mean scores for subgroups of patients with baseline and different numbers of subsequent forms were checked and plotted. If these 'exploratory plots' appeared consistent, we combined the data to examine descriptively patterns of QL over cycles. Median scores were connected through time points to facilitate visual comparison. No paired comparison was performed because of the different patients contributing data at each assessment point. Statistical tests for homogeneity of these plots are not available.

The distribution of patient characteristics in the overall and limited samples was compared using the chi-square test.

The initial prognostic factors were treated as categorical, including gender (female *vs* male), age ($<60$ *vs* $\geqslant 60$ years), initial tumour stage (limited *vs* extensive disease), WHO performance status (0–1 *vs* 2–3) and previous weight loss ($<5\%$ *vs* $\geqslant 5\%$). Serial measures of performance status were not considered. The clinical variables included treatment arm (early *vs* late alternating) and clinical response (partial/complete *vs* no change/progression). Missing QL values were not replaced by assigned values.

All *P* values were derived from two-sided significance tests. Because of the exploratory approach resulting in multiple testing, the interpretation of *P* values changes. Our findings should be confirmed in other studies.

### Results

### Patient characteristics and QL compliance

As detailed elsewhere, the average rate of returned questionnaires with an exact timing over the six cycles was 49% (Hürny *et al.*, 1992). With the extended time window, 54–56% of expected scale scores were available. The subgroup with QL data was compared with the one without and no significant differences were found regarding biomedical data (Bernhard, 1992). The number of evaluable patients/assessments differed between the four scales (Tables I and II). The overall and limited samples had a similar distribution of patient characteristics; the two treatment groups were balanced.

### Overall sample

Levels of initial *tumour stage*, *performance status* and *weight loss* discriminated the average scores of each scale during all cycles. Patients with poor prognostic factors reported worse scores (i.e. higher scores), as shown for emotional well-being (Bf-S) in Figure 1.

The summary measure for 'overall' QL was also predicted in all scales by initial prognostic factors in multivariate analyses (Table I). Older patients reported worse fatigue/malaise. Patients with extensive disease reported worse general and emotional well-being. Those with greater weight loss reported worse fatigue/malaise and general well-being. Those with poor performance status reported worse fatigue/malaise, personal functioning and emotional well-being.

### Limited sample (with baseline QL)

*Baseline QL according to prognostic factors* Baseline differences, according to the level of initial prognostic factors, were present in all scales. Patients with poor prognostic factors reported worse baseline QL scores (Table II; note that the subgroups 'females' and 'performance status 2–3' include very few patients). Significant differences were seen by tumour stage for personal functioning (median values, 0 *vs* 25 for limited *vs* extensive disease) and emotional well-being (median values, 6 *vs* 18 for limited *vs* extensive disease). According to weight loss, there were significant differences for fatigue/malaise (median values, 40

**Table I** Overall sample: patient characteristics and predictive value of prognostic factors for 'overall' QL (multiple regression analyses)[a,b]

| | N *for fatigue/malaise* | *Fatigue/malaise coefficient (s.e.)* (N=*124*; n=*444*)[c] | *Personal functioning coefficient (s.e.)* (N=*130*; n=*459*)[c] | *General well-being (LASA) coefficient (s.e.)* (N=*125*; n=*440*)[c] | *Emotional well-being (Bf-S) coefficient (s.e.)* (N=*127*; n=*453*)[c] |
|---|---|---|---|---|---|
| Sex | | | | | |
| Female | 12 (10%) | – | – | – | – |
| Male | 112 (90%) | −1.46 (2.5) | −1.18 (6.6) | 0.39 (8.10) | 1.28 (3.2) |
| Age (years) | | | | | |
| <60 | 68 (55%) | – | – | – | – |
| ⩾60 | 56 (45%) | **7.26 (1.48)** | 0.82 (3.9) | 0.65 (4.85) | 0.59 (1.94) |
| Tumour stage | | | | | |
| Limited | 41 (33%) | – | – | – | – |
| Extensive | 83 (67%) | 1.83 (1.54) | 7.82 (4.16) | **12.04 (5.06)** | **4.11 (2.04)** |
| Weight loss | | | | | |
| <5% | 81 (65%) | – | – | – | – |
| ⩾5% | 43 (35%) | **7.59 (1.65)** | 5.63 (4.21) | **13.26 (5.14)** | 3.53 (2.05) |
| Performance status | | | | | |
| 0–1 | 107 (87%) | – | – | – | – |
| 2–3 | 17 (13%) | **9.64 (2.31)** | **14.62 (5.59)** | 4.62 (6.94) | **6.4 (2.7)** |

[a]Bold values indicate $P \leqslant 0.05$. [b]Models adjusted for treatment arm, tumour response, language. [c]$N$ = total number of patients; $n$ = total number of assessments.

**Table II** Limited sample: patient characteristic and baseline QL[a]

| | *Fatigue/malaise (0–100)*[b] | | *Personal functioning (0–100)*[b] | | *General well-being (LASA) (0–100)*[b] | | *Emotional well-being (Bf-S) (0–56)*[b] | |
|---|---|---|---|---|---|---|---|---|
| | N | *Median* | N | *Median* | N | *Median* | N | *Median* |
| Sex | | | | | | | | |
| Female | 6 | 57 | 5 | 50 | 4 | 26 | 6 | 14 |
| Male | 43 | 47 | 48 | 17 | 47 | 51 | 45 | 12 |
| Age (years) | | | | | | | | |
| <60 | 30 | 47 | 32 | 17 | 30 | 53 | 31 | 11 |
| ⩾60 | 19 | 47 | 21 | 17 | 21 | 50 | 20 | 20 |
| Tumour stage | | | | | | | | |
| Limited | 19 | 40 | 19 | **0** | 18 | 44 | 20 | **6** |
| Extensive | 30 | 53 | 34 | **25** | 33 | 52 | 31 | **18** |
| Weight loss | | | | | | | | |
| <5% | 29 | **40** | 33 | 17 | 34 | **47** | 32 | 10 |
| ⩾5% | 20 | **60** | 20 | 17 | 17 | **63** | 19 | 13 |
| Performance status | | | | | | | | |
| 0–1 | 42 | **40** | 46 | **17** | 44 | **50** | 45 | **11** |
| 2–3 | 7 | **63** | 7 | **83** | 7 | **100** | 6 | **34** |

[a]Bold values indicate $P \leqslant 0.05$ (Wilcoxon rank-sum test). [b]Full-scale (lower values indicate better QL).

*vs* 60 for <5% *vs* ⩾5% weight loss) and general well-being (median values, 47 *vs* 63 for <5% *vs* ⩾5% weight loss). Performance status had a significant impact on all scales at baseline. The proportion of variance in baseline scores explained by these prognostic factors was 22% for fatigue/malaise and 33–35% for the other scales.

**Figure 1** Overall sample: emotional well-being (Bf-S) over six cycles by levels of initial prognostic factors (±2 s.e.). Average scores are connected through time points to facilitate visual comparisons; they are based on different sample sizes.

*'Minimal', 'maximum' and 'final' improvement in QL under chemotherapy* In the subsample of patients with baseline and at least one subsequent QL assessment, we observed an overall improvement (i.e. subsequent scores were significantly lower than baseline scores) in all scales except for personal functioning, as summarised in Table III (overall; see last row). The improvements were most pronounced in general well-being. The range of improvements is described approximately by the range between MIN and MAX. As an example, the improvement in general well-being ranged between −7 (MIN) and −43 (MAX).

In this group of patients, we compared the three summary measures (MAX, MIN, FIN) by levels of prognostic factors separately for each scale. In general, the improvement was greater in patients with poor prognostic factors (Table III; note that the subgroups 'females' and 'performance status 2–3' include very few patients). In detail, patients with extensive disease showed a significantly greater improvement in personal functioning (MAX = −17) than those with limited disease (MAX = 0). Patients with ⩾5% weight loss showed a significantly larger improvement in fatigue/malaise (MIN = −7) than those with weight loss <5% (MIN = 7) and a much larger improvement in general well-being (see MAX, MIN, FIN in Table III). Patients with poor performance status had a larger improvement in fatigue/malaise (MAX = −13 *vs* −33 for performance status 0–1 *vs* 2–3, in personal functioning (all MAX, MIN, FIN) and emotional well-being (MAX = −5 *vs* −22 for performance status 0–1 *vs* 2–3). Figure 2 shows the maximum improvement of general well-being by weight loss and personal functioning by performance status.

The results of the three summary measures were confirmed by multivariate analyses, as summarised in Table IV for 'maximum' improvement. A different finding concerned weight loss: patients with a previously higher weight loss reported a deterioration in emotional well-being. The predictive value of performance status was confirmed for personal functioning and general and emotional well-being. In addition, male patients reported better general well-being and older patients reported worse general well-being.

*Pattern of QL over cycles (graphical representation)*

In the graphical representation patients with poor initial prognostic factors again showed worse QL. Those with

**Table III** Limited sample: 'minimum', 'maximum' and 'final' improvement in QL during chemotherapy (medians)[a]

| | *Fatigue/malaise (0–100)*[b] N = *49* | | | *Personal functioning (0–100)*[b] N = *53* | | | *General well-being (LASA) (0–100)*[b] N = *51* | | | *Emotional well-being (Bf-S) (0–56)*[b] N = *51* | | |
|---|---|---|---|---|---|---|---|---|---|---|---|---|
| | *MIN* | *MAX* | *FIN* | *MIN* | *MAX* | *FIN* | *MIN* | *MAX* | *FIN* | *MIN* | *MAX* | *FIN* |
| Sex | | | | | | | | | | | | |
| Female | 17 | −23 | −7 | 0 | −50 | −50 | 25 | −8 | 24 | −2 | −6 | −6 |
| Male | 0 | −13 | −7 | 0 | 0 | 0 | −22 | −44 | −36 | 1 | −5 | −3 |
| Age (years) | | | | | | | | | | | | |
| <60 | 0 | −10 | −7 | 0 | 0 | 0 | −26 | −43 | −38 | 1 | −5 | −3 |
| ⩾60 | 0 | −13 | −7 | 0 | −17 | 0 | 0 | −43 | −16 | 0 | −6 | −4 |
| Tumour stage | | | | | | | | | | | | |
| Limited | 0 | −13 | −7 | 0 | **0** | 0 | −2 | −34 | −17 | 1 | −5 | −2 |
| Extensive | 0 | −13 | −7 | 0 | **−17** | −8 | −22 | −45 | −36 | 0 | −6 | −6 |
| Weight loss | | | | | | | | | | | | |
| <5% | **7** | −13 | −7 | 0 | 0 | 0 | **−1** | **−37** | **−15** | 0 | −5 | −3 |
| ⩾5% | **−7** | −20 | −10 | 0 | −8 | −8 | **−36** | **−49** | **−44** | 2 | −6 | −4 |
| Performance status | | | | | | | | | | | | |
| 0–1 | 0 | **−13** | −7 | **0** | **0** | **0** | −5 | −37 | −25 | 0 | **−5** | −3 |
| 2–3 | 7 | **−33** | −27 | **−50** | **−50** | **−50** | −45 | −48 | −45 | −8 | **−22** | −15 |
| Overall[c] | 0 | −13 | **−7** | 0 | **0** | 0 | **−7** | **−43** | **−33** | 0 | **−5** | **−4** |

[a] Bold values indicate $P \leqslant 0.05$ (Wilcoxon rank-sum test); negative values indicate improvement. [b] Full scale range. [c] Bold values indicate $P \leqslant 0.05$ (Wilcoxon signed-rank test).

extensive disease reported worse general well-being over all cycles, as shown in Figure 3a. During the first two cycles only, they reported worse fatigue/malaise and personal functioning, indicating an early and unstable difference. A greater weight loss had an adverse impact on fatigue/malaise, personal functioning and emotional well-being over all cycles (Figure 3b). Similarly, those with poor performance status reported worse scores in all scales over this period, as shown

**Figure 2** Limited sample: box plots of 'maximum' improvement in general well-being (LASA) by level of previous weight loss (**a**) and of personal functioning (PF) by performance status (**b**). The line in the middle of the box represents the median; the box extends from the 25th to the 75th percentile.

**Figure 3** Limited sample: general well-being (LASA) over cycles by levels of initial stage (**a**) and emotional well-being (Bf-S) over cycles by levels of previous weight loss (**b**). Median scores are connected through time points to facilitate visual comparisons; they are based on different sample sizes.

**Table IV** Limited sample: predictive value of prognostic factors for 'maximum' improvement in QL (multiple regression analyses)[a,b]

| | *Fatigue/malaise coefficient (s.e.) (N=48)* | *Personal functioning coefficient (s.e.) (N=52)* | *General well-being (LASA) coefficient (s.e.) (N=50)* | *Emotional well-being (Bf-S) coefficient (s.e.) (N=50)* |
|---|---|---|---|---|
| Sex | | | | |
| Female | – | – | – | – |
| Male | -7.57 (10.52) | -15.71 (12.14) | **-46.98 (19.63)** | 1.21 (5.47) |
| Age (years) | | | | |
| <60 | – | – | – | – |
| ≥60 | 6.32 (7.42) | 2.73 (7.48) | **24.47 (11.20)** | 0.21 (3.90) |
| Tumour stage | | | | |
| Limited | – | – | – | – |
| Extensive | -4.46 (7.11) | -7.45 (7.32) | -4.64 (10.68) | -5.90 (3.65) |
| Weight loss | | | | |
| <5% | – | – | – | – |
| ≥5% | -12.72 (7.69) | 10.10 (8.17) | -14.97 (11.20) | **8.80 (3.79)** |
| Performance status | | | | |
| 0-1 | – | – | – | – |
| 2-3 | 3.69 (10.73) | **-39.80 (11.35)** | **-39.57 (16.59)** | **-13.69 (5.76)** |

[a] Bold values indicate $P \leqslant 0.05$; negative values indicate improvement. [b] Models adjusted for tumour response; fatigue/malaise also for treatment (significant effect).

**Figure 4** Limited sample: general well-being (LASA; **a**) and emotional well-being (Bf-S; **b**) over cycles by levels of performance status. Median scores are connected through time points to facilitate visual comparisons; they are based on different sample sizes.

in Figure 4 for general and emotional well-being. Older patients reported worse fatigue/malaise and general well-being. In addition, they showed a tendency to improve later than younger patients.

In summary, in all three analyses the differential impact of initial tumour stage, weight loss and performance status was consistently confirmed; levels of performance status and weight loss discriminated QL the most. Patients with poor initial prognostic factors reported worse baseline QL and showed a greater improvement under treatment. However, in general, their scores did not reach the values of those with good prognostic factors.

### Discussion

Do initial prognostic factors predict QL during chemotherapy in patients with SCLC? In clinical experience, there is large variability in psychosocial adjustment of lung cancer patients, related to the course of disease and treatment. A positive relationship has been described between QL and good physical functioning as well as limited disease (Ganz *et al.*, 1992).

As expected, in the present study, those patients with poor prognostic factors at diagnosis consistently reported worse QL at baseline than those with good prognostic factors. A similar association between the patients' performance status, extent of disease and psychological distress, as measured by the Profile of Mood States (POMS; McNair *et al.*, 1971), has been reported in a randomised trial including over 400 SCLC patients (Cella *et al.*, 1987). In this study, physical impairment showed an approximately linear relationship to increasing levels of psychological distress at baseline but accounted for a smaller amount of the variability in mood disturbance (10–15%) than the present data.

Correspondingly, given the high response rates, a substantial improvement in QL under chemotherapy is expected, particularly in patients with poor initial prognostic factors. Our findings support this assumption: patients with poor initial prognostic factors showed the most pronounced improvement in QL under cytotoxic treatment. A similar improvement within the first three chemotherapy cycles has recently been shown in another SCLC trial (Wolf *et al.*, 1991). Chemotherapy can be helpful in adjusting to diagnosis, as tumour response to treatment improves physical performance and alleviates symptoms (Bleehen *et al.*, 1993). The question remains, however, whether QL is further improving after response in these patients with extensive disease and resulting in scores similar to those in patients with good prognostic factors.

After controlling for tumour response, and despite the substantial improvement reflected in the change of QL measures, patients with poor initial prognostic factors reported worse scores over cycles and generally did not reach the level of the good risk patients.

In extensive-disease patients with primarily palliative treatment, a cost–benefit estimate between survival/palliation and side-effects of treatment has to be performed. We have recently reported a trial in extensive disease patients comparing our standard regimen of cisplatin, doxorubicin and etoposide alternating with cyclophosphamide, methotrexate, vincristine and lomustine with a mild treatment of carboplatin and teniposide (Joss *et al.*, 1995*a*). Contrary to expectation, patients receiving the latter regimen had a significantly lower remission rate and survival; the trial had therefore to be closed prematurely. In this small trial, physician and patient-rated side-effects were worse with the standard regimen, but no difference was observed in patient-rated tumour symptoms and general aspects of QL.

In a Cancer Research Campaign trial, 300 patients with untreated limited and extensive SCLC and no progressive disease after the first cycle of cytotoxic treatment were randomised to receive cyclophosphamide, vincristine and etoposide either regularly 'planned' or given 'as required' for tumour-related symptoms and progression of disease with a maximum of eight cycles (Earl *et al.*, 1991). Patients receiving treatment 'as required' received on average half as much chemotherapy, but did not show a significantly shorter survival. However, in a subsample of 62 patients with QL assessment, those with treatment as required scored more severe symptoms than those receiving planned treatment. Similar results have been reported in metastatic breast cancer (Coates *et al.*, 1987).

In addition to symptom alleviation, treatment is usually associated with hope and supports particular patients in coping with their anxiety of fatal outcome. However, the optimal balance between treatment intensity, biomedical and QL outcome needs further specification. According to the present knowledge and our data, patients with limited disease benefit subjectively, in terms of their QL, from the currently available, intensive and regularly scheduled, standard chemotherapy regimens, as toxic side-effects are largely outweighed by improvement of tumour-related symptoms and therefore general well-being. These patients should be treated accordingly.

In patients with extensive disease, especially the elderly, the choice of treatment is more difficult. As their QL is substantially improving, mainly during the first three treatment cycles, but does not reach the level observed in limited-disease patients even after response, a conceivable treatment option could be to treat these patients intensively to maximum response and to continue with a milder maintenance therapy including best supportive care. This option has to be tested in a randomised clinical trial against intensive standard chemotherapy, as used for patients with limited disease.

In the present analysis, we have chosen four QL measures sensitive to both disease symptoms and treatment side-effects and giving an overall estimation of patients' physical and psychological well-being (Bernhard, 1992). All were sensitive to initial prognostic factors but showed distinct patterns, which may be explained by their different concepts and time frames. As in other QL trials in SCLC patients (Bleehen *et al.*, 1989, 1993; Gower *et al.*, 1995), missing data limit this analysis, although summary measures revealed consistent findings among related subgroups and concepts. As drop-out rates increased with time, data were collected only from patients with a better health status. Thus, analyses of changes in QL may show, to a certain extent, an attrition bias (i.e. rapidly diminishing patient numbers). In addition, only major differences could be demonstrated owing to the small sample size and related reduced statistical power.

The potential underlying interactions between prognostic factors, treatment, course of disease and patient's adjustment process need further study, including improved methods for handling missing data and longitudinal analyses. Clinically, a high-risk group for poor adjustment is of particular concern, as suggested by a screening trial for psychological morbidity in advanced SCLC (Hopwood and Thatcher, 1990). In addition, patients with poor performance status may find it too burdensome to participate actively in the treatment decision (Blanchard *et al.*, 1988).

In conclusion, initial tumour stage, performance status and previous weight loss can predict QL during chemotherapy, even after controlling for response to treatment. To determine the optimal balance between treatment intensity, efficacy and QL in SCLC remains a challenge. Prognostic factors for psychosocial outcome should be investigated further, possibly providing additional information for the difficult decision of how to treat SCLC patients with extensive disease.

**Acknowledgements**
We gratefully acknowledge the cooperation of our patients, nurses, data managers and physicians in collecting these data.

### References

AARONSON NK, BAKKER W, STEWART AL, VAN DAM FSAM, VAN ZANDWIJK N, YARNOLD JR AND KIRKPATRICK A. (1987). Multidimensional approach to the measurement of quality of life in lung cancer clinical trials. In *The Quality of Life of Cancer Patients*, Aaronson NK and Beckmann J. (eds) pp. 101-109. Raven Press: New York.

BERGMAN B AND AARONSON NK. (1995). Quality-of-life and cost-effectiveness assessment in lung cancer. *Curr. Opin. Oncol.*, **7**, 138-143.

BERNHARD J. (1992). 'Lebensqualität' in Onkologischen Therapiestudien. Konzepte, Methodik und Anwendung am Beispiel des Kleinzelligen Bronchuskarzinoms. Peter Lang: Bern.

BERNHARD J AND GANZ PA. (1995). Psychosocial issues in lung cancer patients. *Cancer Treat. Res.*, **72**, 363-390.

BLANCHARD CG, LABRECQUE MS, RUCKDESCHEL JC AND BLANCHARD EB. (1988). Information and decision-making preferences of hospitalized adult cancer patients. *Soc. Sci. Med.*, **27**, 1139-1145.

BLEEHEN NM, FAYERS PM, GIRLING DJ AND STEPHENS RJ. (1989). Survival, adverse reactions and quality of life during combination chemotherapy compared with selective treatment for small cell lung cancer. *Resp. Med.*, **83**, 51-58.

BLEEHEN NM, GIRLING DJ, MACHIN D AND STEPHENS RJ. (1993). A randomised trial of three or six courses of etoposide cyclophosphamide methotrexate and vincristine or six courses of etoposide and ifosfamide in small cell lung cancer (SCLC). II: Quality of life. Medical Research Council Lung Cancer Working Party. *Br. J. Cancer*, **68**, 1157-1166.

CELLA DF, OROFIAMMA B, HOLLAND JC, SILBERFARB PM, TROSS S, FELDSTEIN M, PERRY M, MAURER LH, COMIS R AND ORAV EJ. (1987). The relationship of psychosocial distress, extent of disease, and performance status in patients with lung cancer. *Cancer*, **60**, 1661-1667.

COATES A, GEBSKI V, BISHOP JF, JEAL PN, WOODS RL, SNYDER R, TATTERSALL MHN, BYRNE M, HARVEY V, GILL G, SIMPSON J, DRUMMOND R, BROWNE J, VAN COOTEN R AND FORBES JF FOR THE AUSTRALIAN-NEW ZEALAND BREAST CANCER TRIALS GROUP. (1987). Improving the quality of life during chemotherapy for advanced breast cancer. A comparison of intermittent and continuous treatment strategies. *N. Engl. J. Med.*, **317**, 1490-1495.

DAVIS CS. (1991). Semi-parametric and non-parametric methods for the analysis of repeated measurements with applications to clinical trials. *Stats. Med.*, **10**, 1959-1980.

EARL HM, RUDD RM, SPIRO SG, ASH CM, JAMES LE, LAW CS, TOBIAS JS, HARPER PG, GEDDES DM, ERAUT D, PARTRIDGE MR AND SOUHAMI RL. (1991). A randomised trial of planned versus as required chemotherapy in small cell lung cancer: a Cancer Research Campaign trial. *Br. J. Cancer*, **64**, 566-572.

EORTC QUALITY OF LIFE STUDY GROUP. (1995). EORTC QLQ-C30 *Scoring Manual*. EORTC Data Center: Brussels.

FLETCHER A, GORE S, JONES D, FITZPATRICK R, SPIEGELHALTER D AND COX D. (1992). Quality of life measures in health care. II. Design, analysis and interpretation. *Br. Med. J.*, **305**, 1145-1148.

GANZ PA, SCHAG CAC, LEE JJ AND SIM MS. (1992). The CARES: a generic measure of health-related quality of life for patients with cancer. *Qual. Life Res.*, **1**, 19-29.

GOWER NH, RUDD RM, DE ELVIRA MCR, SPIRO SG, JAMES LE, HARPER PG AND SOUHAMI RL. (1995). Assessment of 'quality of life' using a daily diary card in a randomised trial of chemotherapy in small-cell lung cancer. *Ann. Oncol.*, **6**, 575-580.

HOPWOOD P AND THATCHER N. (1990). Preliminary experience with quality of life evaluation in patients with lung cancer. *Oncology*, **4**, 158-162.

HOPWOOD P, STEPHENS RJ AND MACHIN D. (1994). Approaches to the analysis of quality of life data: experiences gained from a medical research council lung cancer working party palliative chemotherapy trial. *Qual. Life Res.*, **3**, 339-352.

HÜRNY C, BERNHARD J, JOSS R, WILLEMS Y, CAVALLI F, KISER J, BRUNNER K, FAVRE S, ALBERTO P, GLAUS A, SENN HJ, SCHATZMANN E, GANZ PA AND METZGER U FOR THE SWISS GROUP FOR CLINICAL CANCER RESEARCH (SAKK). (1992). Feasibility of quality of life assessment in a randomized phase III trial of small cell lung cancer - a lesson from the real world. *Ann. Oncol.*, **3**, 825-831.

JOSS RA, ALBERTO P, HÜRNY C, BACCHI M, LEYVRAZ S, THÜRLIMANN B, CERNY T, MARTINELLI G, STAHEL R AND LUDWIG C FOR THE SWISS GROUP FOR CLINICAL CANCER RESEARCH (SAKK). (1995*a*). Quality versus quantity of life in the treatment of patients with advanced small-cell lung cancer? A randomized phase III comparison of weekly carboplatin and teniposide versus cisplatin, adriamycin, etoposide alternating with cyclophosphamide, methotrexate, vincristine and lomustine. *Ann. Oncol.*, **6**, 41-48.

JOSS RA, BACCHI M, HÜRNY C, BERNHARD J, CERNY T, MARTINELLI G, LEYVRAZ S, SENN HJ, STAHEL R, SIEGENTHALER P, LUDWIG C AND ALBERTO P FOR THE SWISS GROUP FOR CLINICAL CANCER RESEARCH (SAKK). (1995*b*). Early versus late alternating chemotherapy in small-cell lung cancer. *Ann. Oncol.*, **6**, 157-166.

MATTHEWS JNS. (1993). A refinement to the analysis of serial data using summary measures. *Stat. Med.*, **12**, 27-37.

MCNAIR DM, LORR M AND DROPPLEMAN LF. (1971). EITS manual for the profile of mood states. Educational and Industrial Testing Service: San Diego.

NETER J, WASSERMAN W AND KUTNER MH. (1985). Applied linear statistical models. Regression, analysis of variance, and experimental designs. Irwin: Illinois.

SIEGEL S. (1956). Nonparametric statistics for the Behavioral Sciences. McGraw-Hill: New York.

TANDON PK. (1990). Application of global statistics in analyzing quality of life data. *Stat. Med.*, **9**, 819-827.

VON ZERSSEN D. (1986). Clinical self-rating scales (CSRS) of the Munich Psychiatric Information System (PSYCHIS München). In *Assessment of Depression*, Sartorius N and Ban TA. (eds) pp. 270-303. Springer: Berlin.

WOLF M, PRITSCH M, DRINGS P, HANS K, SCHROEDER M, FLECHTNER H, HEIM M, HRUSKA D, MENDE S, BECKER H, DANNHÄUSER J, LOHMÜLLER R, GROPP C, GASSEL WD, HOLLE R AND HAVEMANN K. (1991). Cyclic-alternating versus response-oriented chemotherapy in small-cell lung cancer: a German multicenter randomized trial of 321 patients. *J. Clin. Oncol.*, **9**, 614–624.

WORLD HEALTH ORGANIZATION. (1979). *WHO Handbook for Reporting Results of Cancer Treatment*. WHO Offset Publication No. 48: Geneva.